# Functional Limitations and Perceived Neighborhood Walkability Among Urban Dwelling Older Adults

**DOI:** 10.3389/fpubh.2021.675799

**Published:** 2021-06-30

**Authors:** Rie Suzuki, Jennifer Blackwood, Noah J. Webster, Shailee Shah

**Affiliations:** ^1^Department of Public Health and Health Sciences, College of Health Sciences, University of Michigan-Flint, Flint, MI, United States; ^2^Physical Therapy Department, University of Michigan–Flint, Flint, MI, United States; ^3^Institute for Social Research, University of Michigan, Ann Arbor, MI, United States

**Keywords:** limitation of activity, neighborhood, minority, accessibility (for disabled), aged, walkability

## Abstract

Older adults with functional limitations (FLs) often experience obstacles to walking. Although health promotion programs targeting physical activity are available in lower-income areas, few studies have compared the walking experiences of older adults who have FLs with those who do not in the community. The purpose of this cross-sectional survey was to compare perceptions of neighborhood walkability among older adults living in lower-income communities with and without FLs. Participants (*N* = 132) were recruited in 2018 at regional health clinics in Flint, Michigan. To be eligible, participants had to be 65 years of age or older, report no cognitive decline, and be Flint residents. Of the 132 participants, the mean age was 69.74 (SD = 4.97). The majority were female (66%); African American (77%); single, divorced, or widowed (72%); educated below the General Education Development level (57%), and had a FL (67%). Older adults with FLs were significantly (*p* < 0.05) less likely than those without to visit many places within walking distance, to have well-lit neighborhoods at night, and to reside in neighborhoods where sidewalks were separated from the road and traffic. Multiple regression analyses revealed that having a FL was associated with poorer neighborhood perceptions of *mixed-land-use* (*b* = −0.19, *p* < 0.05) and more *walking hazards* (*b* = −0.26, *p* < 0.05). Findings suggest that a FL is associated with perceptions of walkability. It is essential to develop disability-friendly support systems and accommodations to encourage walking in lower-income communities.

## Introduction

The built environment refers to all buildings, spaces, and products which can be created or changed by people (1). The positive built environment is a primary predictor of “*active aging*,” which refers to the process of optimizing opportunities for health, participation, and security that will enhance the quality of life as individuals age (2). Active aging is associated with lower morbidity and mortality rates (3). The Healthy People 2030 framework suggests that improved safety in neighborhoods is necessary to improve health (4). However, research has consistently indicated that because of unsupportive features in their neighborhoods (5), older adults have an increased risk of falls (and fall related injuries), sedentary lifestyles (6), and poor mental health (7).

One of the challenges to achieving “*active aging*” is declining function with age. The World Health Organization (WHO) defines functional limitation (FL) broadly, such that it includes impairments, activity limitations, or participation restriction. Thus, FL entails disruptions to normal movement, vision, hearing, social relationships, and interactions (8). The WHO disability model integrates the pathway between activity limitations (or performance or accomplishment of activity) and environmental contextual factors (e.g., the built environment), both of which contribute to disability incidence (8). The Americans with Disabilities Act Accessibility Guidelines suggests decreasing negative environmental experiences based on the quality of the pedestrian infrastructure and the overall accessibility of destinations (9). Additionally, the literature has reported that a greater fit between older adults' functional health status and the built environment can result in improved quality of life and optimal functioning (7).

Neighborhood walkability is the extent to which neighborhood design supports walking in an area and one of the factors contributing a greater fit between individual function and the built environment (5, 10) The construct is comprised of environmental and social factors such as *mixed land-use* (e.g., a mix of stores and services within walking distance) (11, 12) and the presence of *walking hazards* (e.g., walkable surfaces) (10). A longitudinal study reported associations between these constructs and FLs among older adults. For example, inaccessible public buildings, lack of access to parks, and poor pavement conditions were related to FLs over 3 years (13). Other studies have demonstrated that the relationship between *pedestrian safety* (e.g., less heavy traffic) and FLs are not found in older adults (14, 15). These events may occur because older adults are less likely to walk outdoors if crossing the street safely is perceived as being difficult (16) or if it takes extra time or is difficult to react quickly to a forthcoming danger on the street (6, 17).

The literature has also demonstrated that higher crime rates in one's neighborhood discourage older individuals from walking (18). Older adults' outdoor walking is sensitive to their perceptions of safety because they are generally physically vulnerable if defending themselves against assault on the street (15). Despite these findings, several studies have demonstrated that perceptions of crime and FLs are not related among older adults (7, 15). This finding may reflect the perceived safety from greater policing levels than it reflects underlying crime rates (7). Having witnessed or been the victim of a crime can heighten crime perception (15).

Lower socioeconomic status (SES) in communities has been found to be related to FLs (19). Wealthier neighborhoods provide more resources (e.g., parks, neighbors willing to help each other out) than poorer neighborhoods, resulting in fewer supports that help in maintaining individuals' physical function (20). Conversely, less mixed-land-use in a neighborhood (e.g., lack of stores nearby) may hinder social activities and contribute to disabling conditions (21). This is due to older adults spending the vast majority of their time in their immediate neighborhood (22). Examining how FLs are related to the perceptions of neighborhood characteristics among older adults living in lower SES neighborhoods can help identify modifiable factors that may explain higher rates of FLs among this population.

Previous studies found that residential location, as indicated by zip code, contributes to health conditions among the older adults residing in lower SES communities (23). However, the literature has not examined which environmental factors are negatively involved in older adults with FLs. Additionally, few studies have compared perceptions of walkability in older adults with and without FLs in a lower SES community (24). This study will assess the role of the perceived built environment among older adults residing in a lower SES urban setting to fill these gaps in the literature by examining the association between FLs and the environmental characteristics of individuals residing in a lower SES neighborhood to develop intervention programs to improve all older adults' walking activities.

Based on the cited studies, we propose two hypotheses:

Hypothesis 1 (H1). FLs will be associated with poorer perceptions of mixed land-use and more walking hazards.
Hypothesis 2 (H2). FLs will not be associated with either pedestrian safety or perceived crime.

## Materials and Methods

### Study Design and Recruitment

The Institutional Review Board at the first author's university (HUM00151137) approved this study. This cross-sectional study was conducted from January 2017 to October 2018 in Flint, Michigan, USA. The participants' eligibility criteria were as follows: aged 65 years or older, resided in the Flint area in a home or an apartment, able to communicate in English, and had received medical services at one of the health clinics where recruitment was taking place. Individuals residing in an assisted living facility, nursing home, or other environments where medical care was provided were excluded from the study because they typically receive some assistance in day-to-day mobility. The other exclusion criteria were as follows: not achieving a score >4 on the Mini-Cog (25) or having a serious illness requiring medical care, uncontrolled comorbidity (e.g., end end-stage renal disease, late-stage cancer, congestive heart failure, uncontrolled diabetes, an uncontrolled respiratory disease requiring the use of supplemental oxygen), or an Alzheimer's disease diagnosis or diagnosis of another progressive cognitive impairment because this may limit their ability to complete a self-report survey.

#### Study Procedure

Two trained research assistants recruited potential participants from three medically underinsured health clinics. The participants were first screened by applying the inclusion and exclusion criteria and then led to a quiet room in the clinic to complete a paper survey.

The Mini-Cog was used to screen for dementia, which is is comprised of both a recall (3-item word recall) and executive function item (clock drawing test). For this measure, three words were read from a standardized list to the participant. The participant verbally repeated the words back to the researcher to confirm they had heard them correctly and this was repeated twice. The participant was told to remember the three words. Then, for the clock drawing test, they were given a piece of paper with a large circle on it and were instructed to write the numbers of a clock in the circle and to draw the hands so that the time read “ten past eleven.” After the participant completed this, they were asked to repeat the three words given earlier. One point was given for each word recalled, one point for correctly drawing the numbers on the clock, and one point for drawing the correct time. The maximum score achievable is 5 points (25).

The research assistants spoke with 350 patients at the entrance of the health clinics. Of those patients, 218 were excluded (e.g., 7 scored 4 points or less on the Mini-Cog, 90 were younger than age 65, 10 had a serious acute illness requiring medical care, 63 refused to participate, and 48 had not received medical services at the health clinic). Thus, the final sample included for analysis was 132 participants.

All the participants provided written informed consent. Individuals who completed the survey received an incentive ($25 gift card).

### Measures

Our initial plan was to collect all the variables of the Neighborhood Environment Walkability Scale- Abbreviated NEWS-A (26). However, the two aesthetics variables (e.g., “*many canyons/hillsides in my neighborhood limit the number of routes for traveling from place to place*” and “*the streets in my neighborhood are hilly, making my neighborhood difficulty to walk in*”) and the street connectivity subcontracts (e.g., “*many four-way intersection*s” and “*many alternative routes for getting from place to place)* were excluded because the cognitive testing of a draft questionnaire with five older adults residing in Flint revealed that these variables did not represent their neighborhoods. Hence, four sub-constructs (e.g., *mixed-land-use, walking hazard, pedestrian safety, perceived crime*) of NEWS-A were used.

#### Mixed-Land-Use

We assessed perceptions of neighborhood mixed land-use by examining the extent of agreement to six statements: “I can do most of my shopping at local stores,” “Stores are within easy walking distance of my home,” “There are many places to go within easy walking distance from my home,” “It is easy to walk to a transit stop (bus, train) from my home,” and “The distance between intersections in my neighborhood is usually short (100 yards or less or the length of a football field or less).” The responses comprised *strongly disagree* (1), *disagree* (2), *agree* (3), and *strongly agree* (4). Higher values indicated better perceptions of mixed land use (Cronbach's alpha = 0.76).

#### Walking Hazards

Perceptions of walking hazards were assessed by asking the extent of agreement with six statements about their neighborhood: “there are sidewalks on most of the streets in your neighborhood,” “the sidewalks in your neighborhood well-maintained (paved, even, and not a lot of cracks),” “the bicycle or pedestrian trails in or near your neighborhood are easy to get to,” “the sidewalks are separated by parked cars from the road and traffic in your neighborhood,” “there is a grass or dirt strip that separates the streets from the sidewalks in your neighborhood,” and “your neighborhood streets well lit at night.” The responses comprised *strongly disagree* (1), *disagree* (2), *agree* (3) or *strongly agree* (4). Higher values indicated fewer walking hazards (Cronbach's alpha = 0.74).

#### Pedestrian Safety

We assessed pedestrian safety based on three items: “There is so much traffic on the street where I live that walking in my neighborhood is difficult or unpleasant,” “There is so much traffic on nearby streets that walking in my neighborhood is difficult or unpleasant,” and “There are crosswalks and pedestrian signals to help pedestrians cross busy streets in my neighborhood.” The initial responses comprised *strongly disagree* (1), *disagree* (2), *agree* (3), or *strongly agree* (4). Of three variables, two traffic scales were recoded: *strongly disagree* (4), *disagree* (3), *agree* (2), or *strongly agree* (1). Higher values indicated perceptions of greater pedestrian safety (Cronbach's alpha = 0.78).

#### Perceived Crime

We assessed perceived crime using three items: “There is a high crime rate in my neighborhood,” “The crime rate in my neighborhood makes it unsafe to go on walks during the day,” and “The crime rate in my neighborhood makes it unsafe to go on walks at night.” The initial responses comprised *strongly disagree* (1), *disagree* (2), *agree* (3), or *strongly agree* (4). All three scales were recoded: *strongly disagree* (4), *disagree* (3), *agree* (2), or *strongly agree* (1). Higher values indicated perceptions of fewer perceived crime (Cronbach's alpha = 0.85).

#### Functional Limitations

We assessed FLs by considering a participant's difficulty in performing three activities without using special equipment (27): (1) “walking one-quarter mile;” (2) “climbing 10 steps;” and (3) “stooping, bending, or kneeling.” The original response comprised *limited* (1), *not limited* (2), or *unknown if limited* (3). Based on the previous studies in the United States (28, 29), we coded the responses as *limited* (1) if a respondent reported any difficulty with one or more functional activities or *not limited* (0) if a respondent reported no difficulty.

#### General Health

Based on a prevoius study that examined individual walkability (30), we included falls history in the past year prior to the survey, the presence of sensory impairments (vision problems or numbness in the feet) and medication use (taking >3 medications per day). The responses comprised *yes* (1) or *no* (0). Higher scores on all variables indicated a condition's presence.

### Statistical Analysis

Descriptive analyses, chi-square, and analysis of variance were conducted using SAS 9.4. (31). The frequencies, means, and standard deviations were calculated for neighborhood walkability measures, demographic characteristics, and other variables, stratified by FLs (Table 1). Multiple regression analyses were conducted using Mplus 8.4. (32). Assumptions for multiple linear regression analyses were examined using scatterplots (homoscedasticity), histograms (residuals), and the Spearman correlation analysis (multicollinearity). The examination of skewness and kurtosis indicated that the data were not normally distributed; thus, we used the maximum likelihood estimator to reduce the estimated error. Because the Spearman correlation analysis revealed that several predictors were lineally correlated (Table 2), the residual variances among these correlated predictors were allowed to co-vary in the multiple regression models. No missing cases were observed in the data. A *p*-value of < 0.05 was considered statistically significant.

**Table 1:** Characteristics of participants.

	**Total** ***N* = 132**	**With** ** FLs** ***N* = 88**	**Without** ** FLs** ***N* = 44**	***p-*value**
Age in year, mean (SD)	69.74 (4.97)	70.08 (5.39)	69.43 (4.35)	ns
Gender, female	66%	67%	52%	ns
Race, African-American	77%	76%	80%	ns
Marital Status, divorced or separated	72%	73%	70%	ns
Education level, less than high school	57%	54%	65%	ns
Income, receiving SSDI	42%	43%	42%	ns
Assisted device use (Y)	32%	41%	23%	**0.021**
**Health conditions**				
Fall in the past year (Y)	42%	53%	32%	**0.001**
Vision problems (Y)	43%	40%	46%	ns
Numbness in feet (Y)	38%	52%	24%	**0.002**
Medication, > 3/day (Y)	71%	84%	59%	**0.001**
Body mass index, kg/m^2^, mean (SD)	30.73(7.65)	31.54	28.97	ns
Mixed land use ^*^	2.39 (0.86)	2.26	2.67	**0.013**
Walking hazards^*^	2.48 (0.79)	2.37	2.72	**0.018**
Pedestrian safety^*^	2.21 (0.92)	2.33	1.94	**0.024**
Perceived crime^*^	2.31 (0.18)	2.38	2.15	ns

*FL, functional limitation; SD, standard deviation; SSDI, social security disability insurance; Y, yes; ns, not statistically significant at p = 0.05*.

* *Higher scores of neighborhoods walkability indicated the perception of greater mixed-land-use, fewer walking hazards, greater pedestrian safety and little crime. Bold values indicates a statistically significant level at p = 0.05*.

**Table 2:** Spearman correlation matrix.

	**1**	**2**	**3**	**4**	**5**	**6**	**7**	**8**	**9**	**10**
1. Mixed land use^**^	1.00	0.38^*^	0.15	0.02	–0.28^*^	–0.20^*^	−012	−0.15	0.00	−0.08
2. Walking hazard^**^		1.00	0.04	0.14	–0.21^*^	0.05	−0.16	0.06	0.13	0.07
3. Pedestrian safety^**^			1.00	0.34^*^	–0.20^*^	−0.09	−0.04	−0.17	−0.10	0.10
4 Perceived crime^**^				1.00	−0.10	0.06	0.10	−0.15	−0.12	−0.10
5. FL					1.00	0.21^*^	−0.05	0.26^*^	0.27^*^	0.16
6 Fall in the past year						1.00	0.13	0.32^*^	0.23^*^	0.34^*^
7 Vision problems							1.00	0.11	0.11	0.03
8 Numbness in Feet								1.00	0.12	0.29^*^
9.Medication, > 3/day									1.00	0.21^*^
10. Body mass index										1.00
Skewedness	0.25	−0.02	−0.14	−0.18	−0.82	0.19	0.42	0.36	−1.15	0.99
Kurtosis	−0.90	−0.71	−0.94	−1.29	−1.35	−2.00	−1.85	−1.90	−0.67	1.28

*The demographic variables did not indicate the statistically significant relationships with the dependent variables at p = 0.05 level; SD, Standard deviation; FL, functional limitation;*

* *a statistically significant level at p = 0.05;*

** *higher scores of neighborhoods walkability indicated the perception of greater mixed-land-use, fewer walking hazards, greater pedestrian safety and little crime. Bold values indicates a statistically significant level at p = 0.05*.

## Results

### Participant Characteristics

The information in Table 1 indicates that most participants were African American (77%), had less than a high school education (57%), and were divorced or separated (72%). Almost a majority of the participants received social security disability insurance (42%). The mean (M) age was 69.74 years (standard deviation (SD) = 4.79). Group comparisons indicated non-significant differences in demographic variables between the participants with and without FLs; however, there were significant differences in health conditions. Older adults with FLs were more likely to have experienced a fall in the past year, take >3 medications per day, use an assistive device, and report numbness in the feet than those without FLs (*p* < 0.05).

### H1. FL, Mixed-Land-Use, and Walking Hazard

The analysis of variance for the binary variables was used to detect group differences on each variable. The results indicated that older adults with FLs were more likely than those without to have poorer perceptions of mixed land use. For example, those with FLs disagreed that there were many places within walking distance from home (FLs: *M* = 1.91, *SD* = 1.09 vs. without FLs: *M* = 2.45, *SD* = 1.24; *p* < 0.05), and that it was easy to walk to a transit station from home (FLs: *M* = 2.22, *SD* = 1.25 vs. without FLs: *M* = 2.97, *SD* = 1.27; *p* < 0.05). Also, compared to older adults without FLs, those with FLs perceived more walking hazards. For example, those with FLs disagreed that neighborhoods were well-lit at night (FLs: *M* = 2.43, *SD* = 1.22 vs. without FLs: *M* = 2.97, *SD* = 1.22; *p* < 0.05*)* and sidewalks were separated by parked cars from the road and traffic (FLs: *M* = 2.01, *SD* = 1.15 vs. without FLs: *M* = 2.58, *SD* = 1.20; *p* < 0.05).

The zero-order correlation analysis revealed that FLs were associated with perceptions of poorer *mixed*-*land use* (*r* = 0.28, *p* < 0.05), more *walking hazards (r* = 0.21, *p* < 0.05*)* and the presence of greater *pedestrian safety* (*r* = 0.20, *p* < 0.05). Fall experience was also correlated with the presence of poorer perceptions of *mixed*-*land use* (*r* = −0.20, *p* < 0.05) and FL (*r* = 0.21, *p* < 0.05). However, 4 other characteristics linking to older adults' walkability (30)— vision problems, foot numbness, medication use, and BMI—were not associated with these summative variables (Table 2).

Multiple regression analysis examined the relationships between FLs and perceived components of the built environment. According to the information in Table 3, after controlling for fall experience, medication use, and foot numbness, having a FL was associated with perceptions of poorer *mixed*-*land use* (*b* = −0.19., *p* < 0.05, *R*^2^ = 11%) and more *walking hazards* (*b* = −0.26, *p* < 0.05, *R*^2^ = 13%).

**Table 3:** Multivariate regression analysis to predict NEWS subscales after controlling for covariates.

	***Dependent variables***			
	**Mixed land use**	**Walking hazard**	**Pedestrian safety**	**Perceived crime**
	**Beta (SE)**	**Beta (SE)**	**Beta(SE)**	**Beta (SE)**
Functional limitation (Y)	–0.19 (0.09)^*^	–0.26 (0.09)^*^	−0.14 (0.10)	−0.06 (0.09)
**Covariates**				
Fall in the past year	−0.14 (0.09)	0.04 (0.11)	0.06 (0.10)	0.17 (0.10)
Numbness in feet	−0.06 (0.09)	−0.06 (0.10)	−0.11 (0.10)	−0.16 (0.11)
Medication, >3/day	0.11 (0.09)	0.19 (0.08)^*^	−0.02 (0.09)	−0.10 (0.10)
*R^2^*	11%	13%	9%	7%

*Higher scores of neighborhoods walkability indicated the perception of greater mixed-land-use, fewer walking hazards, greater pedestrian safety and little crime. Bold values indicates a statistically significant level at p = 0.05*.

### H2. FL, Pedestrian Safety, and Perceived Crime

Descriptive statistics, zero-order correlation analysis, and multiple regression analyses did not demonstrate relationships between FL and *pedestrian safety* or *perceived crime* (Table 3).

### Covariates

In the multiple regression analysis models (Table 3), medication use was associated with fewer perceived *walking hazards* (*b* = 0.19, *p* < 0.05, *R*^2^ = 13%). However, fall experience and foot numbness were not associated with the self-reported neighborhood walkability in the sample.

*Post hoc* power analysis was conducted by using G power (33). The results indicated that our sample size (*N* = 132) and the number of predictors (*n* = 4) reached a power of.80 to reject the null hypothesis.

## Discussion

Identifying the influence of having a FL on older adults' perceptions of the built environment can potentially enhance the understanding of daily walking behavior in this population. By analyzing data from a convenience sample collected from regional outpatient clinics, we demonstrated that older adults with FLs perceive that they live in neighborhoods with less mixed land-use and more walking hazards. We also demonstrated that having a FL was not associated with perceptions of *pedestrian safety* and *crime*, despite the fact that physical function differed in those with and without FLs.

As hypothesized, our results reconfirm that FL status was associated with the perceptions of less *mixed-land-use* and greater *walking hazards*. This result is consistent with previous research performed using large national data sets indicating that individuals with FLs experienced inaccessibility and worsening health conditions. For example, barrier to sidewalks and pedestrian amenities have been shown to be related to obesity and diabetes (34) and decrease mobility in older adults (35). Our study suggests that older adults without FLs perceived barriers to taking a walk in the community. Those with FLs perceived stronger obstacles to walking around in their neighborhoods. Further research is necessary to confirm whether these environmental features interfere with physical and social activities among individuals with and without FLs who reside in lower SES communities in a nationally representative sample.

Our results demonstrated that FLs were not associated with perceptions of pedestrian safety. Another study found that older adults perceive heavy traffic as a barrier to taking a walk (36). We expanded on that research by focusing on FL status in older adults and found that this built environmental feature was not limiting for older adults residing in a lower SES community with FLs compared to those without. Our findings differed from that of other studies; that is, navigating busy streets was frequently reported as a negative experience (e.g., unable to cross the street on time) among older adults (37). Improving access to public transportation is particularly important to older adults who do not reside in higher SES neighborhoods and have limited or no access to private transportation options. In the city of Flint, older residents might not take a walk in their neighborhoods because they are more likely to experience one or more FLs (37.2%) than their state and national counterparts (35.5%) (38). Additionally, few destinations are available (e.g., insufficient number of grocery stores) in the community (38). Assessing the availability and proximity of community resources such as covered transit stops and stores is important when considering a community's walkability, especially for individuals who walk outdoors as a mode of transportation (14). Developing intervention programs requires securing a safe space near areas with mixed-land-use by providing many destinations and user-friendly environments (e.g., benches and covered areas on pathways to destinations). Further research is necessary to examine how older adults use these modes of transportation to understand their walking opportunities in a community.

Our study also found that among older adults, FL status was not associated with *perceived crime* as a walkability component. Typically, residing in a high crime area can be stressful for older adults with FLs, because of the wide differences between neighborhood safety levels and an individual's ability to physically respond to outer threats. This finding could suggest that the more crime that older adults with FLs perceive in the community, the more they use transportation or door-to-door services. Attitudes toward a neighborhood's walkability and support systems should be assessed to identify the factors associated with walking.

This study's findings support the creation of targeted programs to improve walking as a mode of physical activity which may fulfill a real need within lower SES urban communities because leisure-time physical activity in older adults is influenced by SES (13). Unmaintained external environments such as the lack of resting places, high curbs, poor street conditions, dangerous crossroads, and lack of pedestrian zones create barriers for physical activity (39) decreasing walking opportunities in older adults. Future intervention programs by the multidisciplinary team members (e.g., health professionals, social workers, community health educators, and policymakers) should target the socio-ecological nature of walking among all older adults.

### Limitations

In this study, we assessed correlations between FL status and and perceptions of the built environment of neighborhoods. The results suggest that the availability of safe, walkable routes may be valuable. However, our study has several notable limitations. First, because of this study's cross-sectional nature, the findings could not identify causal links; thus, we could not explain the reasons for the differences in the prevalence of the perceived built environment between older adults with and without FLs. Second, the data was comprised of self-reported responses that are prone to distortion by social desirability and recall. Third, this study did not objectively measure a respondent's built environment. Fourth, the convenient sampling limited the generalization of the findings to lower SES communities in one area, and not in other areas of the world where the urban built environment may differ. Fifth, this study did not identify what aspects of functional limitation were most associated with the features of walkable neighborhoods. Lastly, this study assessed one geographical region with a recent public health environmental crisis, which may have influenced participants' responses to the health and wellness items and their willingness to participate in the survey.

## Conclusions

Our data demonstrated that older adults with FLs experienced inaccessibility and walking hazards. These findings suggest that intervention programs are necessary to educate and support older adults with FLs and healthcare providers to assess their neighborhoods. This improved understanding of the community-level barriers that older adults experience when endeavoring to take a walk can increase the precision in targeting intervention opportunities; for example, community health education plans should include healthy walking practices. Furthermore, refining knowledge among policymakers on the impact of community layouts and available provisions could enhance walking rates. Many older adults use assistive devices (e.g., cane, walker, grab bar, shower seat) or receive help from others to overcome limitations (40). Additional practices may facilitate their ability to take a walk, such as using supportive equipment (e.g., park benches), developing safe walking routes, and adopting flexible scheduling practices.

This study also found that internal falls risk factors (e.g., medication use) were associated with external risk factors (e.g., walking hazards) in older adults who reside in a lower SES community. In clinical settings, neighborhood walkability should be considered a part of the management of falls risk for older adults living in urban settings, and health care providers should ask about how easy they believe it would be to walk to a store or transit stop from their residence. If they indicate the difficulty of walking to either of those locations, fall prevention education should be provided because the risk of falling is greater. Further, when walking is included as a part of a structured exercise program, health care providers should discuss any environmental barriers that may prevent older adults from walking in their neighborhood.

## Data Availability Statement

The datasets generated for this article are not readily available because the IRB did not approve the data sharing. Requests to access the datasets should be directed to rsuzuki@umich.edu.

## Ethics Statement

The studies involving human participants were reviewed and approved by the Institutional Review Board at the University of Michigan (HUM00151137). The patients/participants provided their written informed consent to participate in this study.

## Author Contributions

RS designed the model, the computational framework and analyzed the data. RS and JB carried out the survey recruitment. RS, JB, NW, and SS contributed to the interpretation of the results, provided critical feedback, and helped shape the research, analysis, and manuscript. All authors contributed to the article and approved the submitted version.

## Conflict of Interest

The authors declare that the research was conducted in the absence of any commercial or financial relationships that could be construed as a potential conflict of interest.
